# Evaluating Future Thinking Priming Effects on Delay Discounting Among Young Adult Drinkers

**DOI:** 10.21500/20112084.7551

**Published:** 2025-11-20

**Authors:** Frida García-Rangel, Hugo Reyes-Huerta, Cristiano V. dos Santos, Rodrigo Sosa, Kalina Martínez

**Affiliations:** 1 Universidad Autónoma de Aguascalientes. Aguascalientes, México. Universidad Autónoma de Aguascalientes Universidad Autónoma de Aguascalientes Aguascalientes Mexico; 2 Universidad de Guadalajara. Guadalajara, México. Universidad de Guadalajara Universidad de Guadalajara Guadalajara Mexico; 3 Universidad Panamericana. Guadalajara, México. Universidad Panamericana Universidad Panamericana Guadalajara Mexico

**Keywords:** FTP, delay discounting, binge drinking, priming effect, FTP, descuento por demora, abuso de alcohol, efecto de priming

## Abstract

The extent to which an individual prefers smaller immediate rewards over larger delayed rewards is referred to as delay discounting. Higher discounting is associated with addictive behaviors and reducing it may aid in their prevention or treatment. Consequently, interventions that decrease discounting-such as Future Thinking Priming (FTP)-have attracted research interest; however, the literature on young adults with binge drinking remains scarce. This study aimed to evaluate the effect of Future Thinking Priming (FTP) on delay discounting among individuals who engage in binge and low-risk drinking. We assessed delay discounting in 86 participants divided into two groups (future-oriented priming or control), using a pretest-posttest design. The results did not provide evidence that FTP reduces delay discounting in this sample. This null effect may reflect the fact that successful priming interventions are typically detected with large sample sizes, underscoring the need to examine potential moderators such as self-effcacy and affective valence. Finally, we found a strong correlation between pretest and posttest discounting measures, consistent with studies characterizing delay discounting as a trait variable.

## Resumen.

El grado en que un individuo prefiere recompensas inmediatas más pequeñas sobre recompensas mayores demoradas se denomina descuento por demora. Un mayor descuento se asocia con conductas adictivas, y reducirlo puede ayudar en su prevención o tratamiento. En consecuencia, intervenciones que disminuyen el descuento -como el Future Thinking Priming (FTP)- han atraído interés en las investigaciones; sin embargo, la literatura en adultos jóvenes con consumo excesivo de alcohol sigue siendo escasa. Este estudio tuvo como objetivo evaluar el efecto del Future Thinking Priming (FTP) sobre el descuento por demora en individuos con consumo de alcohol excesivo y de bajo riesgo. Evaluamos el descuento por demora en 86 participantes, divididos en dos grupos (priming orientado al futuro o control), utilizando un diseño pretest-postest. Los resultados no proporcionaron evidencia de que el FTP reduzca el descuento por demora en esta muestra. Este efecto nulo puede reflejar el hecho de que las manipulaciones de priming exitosas se detectan típicamente con tamaños de muestra grandes, lo que indica la necesidad de examinar posibles moderadores como la autoeficacia y la valencia afectiva. Finalmente, encontramos una fuerte correlación entre las medidas de descuento pretest y postest, consistente con estudios que caracterizan el descuento por demora como una variable de rasgo.

## 1. Introduction

The growing global trend in alcohol consumption is a significant concern due to its adverse effects on both physical and mental health. Alcohol abuse accounts for approximately three million deaths annually and is associated with over two hundred diseases and mental disorders (OMS, 2022). While alcohol may initially induce feelings of well-being, euphoria and disinhibition, long- term consumption yields or leads to dependence and contributes to the development of various cancers, cardiovascular diseases, depression, anxiety, and other mental health issues (Müller et al., 2023). One of the reasons why people choose to consume alcohol despite its negative impact on health may be the fact that this negative impact occurs in the future and is probabilistic, while the effects of well-being and euphoria are relatively immediate and certain (Kirby & Petry, 2004; Rung et al., 2019). In this context, al cohol consumption represents an intertem poral choice, a choice between immediate rewards (i.e., well-being and euphoria) and delayed rewards (i.e., optimal health).

Intertemporal preferences are underlain by delay discounting, a process whereby the value of a reward decreases as the delay to its delivery increases (Athamneh et al., 2021). Discount rates are lower when there is a stronger preference for delayed rewards. On the other hand, a greater preference for immediate rewards results in higher discount rates (Rung et al., 2019). Delay discounting has been examined across various species (Vanderveldt et al., 2016) with gains and losses (see Furrebøe, 2022) and using diver se types of rewards, both hypothetical and real, including money, food, substances, and outcomes related to health, or sexual behavior (see Odum et al., 2020). Some studies characterize this variable as a trait given the stability of its measures over time (Rung et al., 2019), while others view it as a state va riable, given its susceptibility to be modified by external factors (e.g., Yi et al., 2008). Recently, a growing body of research defines delay discounting as a transdiagnostic process implicated in numerous impulsivity-related pathologies, including addiction (Rung et al., 2019; Weinsztok et al., 2021).

Substance abuse has been widely associated with a stronger preference for immediate rewards (Amlung et al., 2017; MacKillop et al., 2011). In the case of alcohol, studies have found that alcohol consumers exhibit steeper discounting rates compared to non-consumers (Murphy & Garavan, 2011) and some research proposes that the greter the severity of addiction, the greater the discounting of future rewards (Acuff et al., 2023; Kraplin et al., 2020). Comparable findings have been reported for users of other substances such as tobacco (DeHart et al., 2020), cannabis (Strickland et al., 2021), cocaine (Cox et al., 2020) , and opioids (Peck et al., 2022). Likewise, some studies suggest that delay discounting predicts the risk of relapse (González-Roz et al., 2019; Sheffer et al., 2012), and the onset and development of addiction (Frohner et al., 2022; Fernie et al., 2013). In light of this evidence, the importance of identifying interventions that re duce discounting in consumers has been increasingly recognized, as such interventions may foster a stronger preference for future rewards, including more controlled drinking or, in some cases, total abstinence (Rung et al., 2019). Systematic reviews have suggested that interventions with future-oriented stimuli are particularly effective in reducing delay discounting (Rung & Madden, 2018; Scholten et al., 2019), specifically when the stimuli are directly related to participants and their per sonal experiences (Olsen et al., 2024).

One of these interventions is the Future Thinking Priming (FTP). In general terms, a priming effect occurs when exposure to a specific stimulus predictably influences the response to a following stimulus (Sheffer et al., 2016). In FTP, the stimuli consist of words related to the future, which participants use to construct self-referential sentences. For example, if the word is future, the participant must incorporate it into a personal, self - referential sentence (e.g., “I am excited about my future”; “Me and my friends are excited about the future”).

Future Thinking Priming (FTP) shares features with Episodic Future Thinking (EFT), the primary future-oriented intervention. The key distinction is that, in EFT, participants do not generate self-referential sentences. Instead, they construct specific and detailed future events that they expect to experience on a given date, and these events are subsequently transformed into cues (written or auditory) that are generally presented before or during the delay discounting task (Olsen et al., 2024; Brown & Stein, 2022). To maximize its effect, EFT requires the imagined event to be highly vivid and detailed, including aspects such as who is present, where the per- son is, how they feel, etc. (Olsen et al., 2024; Brown & Stein, 2022). As a result, variation in vividness can constrain the effectiveness of EFT (Brown & Stein, 2022). In this regard, FTP offers several advantages: its effect is independent of stimulus vividness, it requires only minimal attentional resources, and it can be implemented rapidly and delivered online, thereby facilitating large-scale applications (Shevorykin et al., 2019).

Two key studies reported significant reductions in discounting rates as a result of FTP. In a study by Sheffer et al. (2016), participants exposed to future-oriented words exhibited lower discounting rates compared to participants in two control groups. The second study by Shevorykin et al. (2019), included pre- and post-test measures of discounting along with an additional task to measure it (i.e., MCQ and 5-Trial task). This study not only replicated the findings of the first, but also found a significant reduction in discounting between the pre- and post-test measures for future-oriented participants.

The most recent study by Shevorykin et al. (2021) involved a secondary analysis of data from the second study. This analysis examined tobacco consumption as a moderator of FTP's effects on participants. Participants were categorized into two groups: smokers (i.e., those who smoked at least one cigarette per day) and nonsmokers (i.e., those who did not smoke). Application of FTP resulted in a reduction of discounting rates across both groups of participants, with a more pronounced effect observed in smokers compared to nonsmokers. These findings suggest that FTP could be a potent intervention to modify discounting rates in individuals with substance use or abuse.

Although evidence suggests that FTP may reduce delay discounting in substance users, this relationship may be challenged by two key aspects. First, the limited number of studies evaluating this intervention raises questions about the generalizability of its effects. Second, in the two available empirical studies, participants reported alcohol consumption levels above the binge drinking threshold (i.e., consuming more than 4 drinks on a single occasion for women and more than 5 drinks for men; Centers for Disease Control and Prevention [CDC], 2025). Consequently, the effects of FTP may vary among individuals, particularly young adults, who engage in binge drinking. Thus, its generalizability for reducing reward discounting in these drinking patterns and this population remains unclear.

The aim of this study is to evaluate the effects of the Future Thinking Priming (FTP) among individuals engaging in binge and low-risk drinking. If FTP has an effect, the results will provide additional evidence supporting its potential as a tool for facilitating the preference for future rewards in alcohol consumers. If we fail to detect an effect of FTP, this study will serve as a comparison that highlights differing results from previous findings. Such differentiation is essential for identifying which interventions are genuinely effective in reducing de- lay discounting among consumers.

## 2. Method

### 2.1 Participants

We implemented a statistical power analysis in R using the PowerUpR package and its function 'mrss.ira ()' (minimally required sample size; Dong & Maynard, 2013) to de termine the minimum sample required for the effect size of the intervention. We used the main model with parameters: a = 0.05 for statistical significance; n = 0.80 as statistical power criterion; two-tailed criterion for positive or negative differences; Cohen's *d* = -0.34 for the minimum detectable FTP's effect size; *g* = 1 as intrasubject trait covariate; r2 = 0.68 as proportion of variance by the covariate and *p* = 0.50 for the proportion of participants in the future-oriented group. The minimum number of participants was 86.

A total of 96 individuals were recruited, but data from 10 participants were excluded: six due to study dropout and four due to programming errors in the experimental tasks. The final sample comprised 86 participants (48 women and 38 men), all aged between 18 and 25 years (M = 21), who were students or graduates from the Autonomous University of Aguascalientes, a public university in Mexi- co. Participants were recruited via announcements placed across the university campus and nearby areas. The announcements aimed to recruit individuals who consume alcohol to participate in a decision-making study. The primary inclusion criterion was a mini- mum alcohol consumption of twice a month. Exclusion criteria included having a native language other than Spanish, dependence on substances other than alcohol, and a be- low-average intelligence score as assessed by an intelligence scale. Participants were categorized based on their AUDIT scores: those scoring above 8 were classified as binge drinkers, and those scoring 7 or below as low-risk drinkers. This classification followed the cut-off score of the AUDIT (i.e., 8) distinguishing low-risk from binge or heavy drinking. Before the study began, participants signed an informed consent form that outlined the research objective, procedures, study duration, data confidentiality, and the voluntary nature of participation. The Bioethics Committee of the Autonomous University of Aguascalientes approved all recruitment and evaluation procedures.

### 2.2 Instruments

Alcohol consumption was assessed using the Alcohol Use Disorders Identification Test (AUDIT; Cronbach a = 0.84; Quintero et al., 2019). The total score provides an index of risk associated with alcohol use: scores greater than 8 correspond to binge or heavy drinking, while scores of 7 or below correspond to low-risk drinking.

We also applied the Alcohol, Smoking and Substance Involvement Screening Test (AS- SIST) to identify patterns of use for substances other than alcohol (Cronbach a = 0.87; Sainz et al., 2016). The sum score derived from this questionnaire indicates the level of risk associated with substance use: a score of 0 to 3 signifies low-risk, 4 to 26 moderate risk, and 27 or above possible dependence.

To assess intelligence, we employed the Mexican standardized version of the SHIPLEY-2 scale, using the Vocabulary and Abstraction subtests (Cronbach a = 0.87; Shipley et al., 2014). The final standard score provides an estimate of intelligence: below 70 indicates low intelligence, 70 to 79 well below average, 80 to 89 below average, 90 to 109 average, 110 to 119 above average, 120 to 129 well above average, and 130 or above superior intelligence.

There is some evidence that the level of individuals' adjustment to social influences (i.e., self-monitoring) moderates priming effects (Sheffer et al., 2016). Based on this, we applied the Self-Monitoring Scale (r = 0.83; Snyder, 1974) that categorizes self-monitoring levels as follows: 0 to 8 low self-monitoring, 9 to 14 medium self-monitoring, and 15 to 25 high self-monitoring. We used the Spanish-translated version previously applied in Mexican population (see Hosch and Marchioni, 1986).

We also administered a sociodemographic questionnaire collecting information about age, sex, ethnic group, marital status, educational level, monthly familiar income, and country of origin. Finally, we assessed delay discounting with a computerized intertemporal choice task involving hypothetical monetary rewards. Regarding FTP, we used a computerized version of the task implemented in Shevorykin et al. (2019). Both delay discounting task and FTP were presented in touch-screen desktop computers.

### 2.3 Delay Discounting Task

We evaluated how time affects the value of a hypothetical reward of $8000. This stan dard reward could be obtained after a delay in the next order: 1 month, 6 months, 1 year, 2 years, and 4 years. At each delay, the standard reward was presented during a total of eight choices against an immediate reward to identify five indifference points (IP) to reflect the degree of discounting. Every choice was displayed on the screen of the computer as follows:

*"Receiving $8,000 if you wait 1 month or receiving $4,000 now. Which one do you prefer?"*

To obtain an IP, we employed an adjusting-amount procedure (Du et al., 2002), in which the amount of the immediate reward was modified based on the participant's choices, while the delayed reward remained cons- tant. For example, if a participant selected the $8000 in 1 month over the $4000 now, the immediate reward increased by half (50%) of the difference between the two amounts of reward for the next choice (i.e., $2000). As a result, in the second trial the choice would be between $6000 now and $8000 in 1 month. Conversely, if in the first trial the participant selected $4000 now over the $8000 in 1 month, the immediate reward for the next choice decreased by half (50%) of the difference between the two rewards. In this case, in the second trial choice would be between $2000 now and $8000 in 1 month. For the subsequent choices, the percentage of the adjustment diminished to half of the preceding one (i.e., 25%, 12.5%, 6.25%, 3.12%, 1.56%, 0.78%). These adjustments were indicated by three brief flickers in the amount of money offered at the beginning of the choices. The adjustments persisted until the eighth choice and then restarted for the subsequent choices of the following delay. The change in delay was communicated to participants through the following message displayed at the center of the screen:

*“Please note that the delay for receiving $8,000 has changed."*

Participants were first presented with the general task instructions (see Appendix A) and then they were allowed to begin. To indicate their choice, they were required to drag the preferred reward and its corresponding delay into designated preferred and non-preferred boxes displayed on the screen. Participants were not allowed to proceed to the next choice unless the selected amounts and delays were properly matched.

### 2.4 Future Thinking Priming Task (FTP)

After providing their identification data and reading the general instructions of the task, participants received a list of ten words corresponding to their group (i.e., future-oriented or control). Each word appeared one by one in the center of the screen, with black letters and white background. In both groups the words were the same as in Shevorykin et al. (2019) but translated from English to Spanish by Spanish speakers with English as their native language. For participants in the future-oriented group the words were: *future, self-discipline, willpower, discipli ne, restraint, self-control, long-term, save, planned and investment*. For participants in the control group the words were: *pale, drab, informative, patriotic, detached, dispassionate, middle of the road, disinterested, loud and formal*. Participants were required to create 10 self-referential sentences using those words, along with a paragraph describing themselves. Emphasis was placed on ensuring that the sentences and paragraph referred only to themselves and not to others.

All words were presented to participants in the same order, with sentence instructions displayed immediately afterward. Once participants completed their sentences, instructions for the paragraph appeared, followed by a designated space for writing. To see the specific instructions, refer to Appendix B.

### 2.5 Procedure

We implemented a procedure similar to the one described by Shevorykin et al. (2019). Therefore, this study comprised two sessions, each lasting approximately one hour and separated by two weeks. In the first session, participants signed the informed consent form and completed the AUDIT, ASSIST, SHIPLEY-2, Self-Monitoring Scale, and sociodemographic questionnaire. After completing these tests, we conducted the pretest assessment of delay discounting. After the first session, participants were assigned to either the future-oriented or control group. Participants were matched according to their type of alcohol consumption, the number of substances consumed other than al cohol, and their intelligence score. In the second session, we administered the Future Thinking Priming Task (FTP), followed by the posttest measurement of delay discounting. Both sessions were conducted individually in a well-lit room.

### 2.6 Data Analysis

To estimate delay discounting rates, we calculated the Area Under the Curve (AUC) for each function as follows:




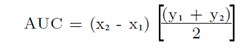




where Xi and X_2_ are the successive delay va lúes and Yi and Y2 the indifference points associated with those values. Values close to 0 indicate higher discounting and those close to 1 indicate lower discounting.

We assessed systematicity of delay discounting data using Johnson & Bickel (2008) criteria. Delay discounting functions are nonsystematic when (1) the value of the reward increases or (2) does not decrease over time. To identify them, we applied Criterion 1 (i.e., indifference points that exceeded the previous one by 20% of the delayed amount) and Criterion 2 (i.e., the indifference point of last delay was not lower than the indifference point of the first delay by at least 10% of the delayed amount).

For the differences between the pre- and post test AUC means of both groups, we created two linear mixed regression models (LMRM) using R Studio, with the ngme package and its 'ngme()' function (Asar et al., 2020). The main model was Phase [Pre - Post] X Group [FTP, CTL] + intrasubject traits as a random intercept. For the exploratory analysis of alco hol consumption, the model was: Phase [Pre - Post] X Group [FTP, CTL] X Alcohol + intrasubject traits. We report Beta regression coeffcient (3), 95 % confidence intervals (C.I.) and the statistical significance value (*p*) of each model. We omitted the detailed description of the models including the other variables as none of them proved significant correlation with AUC. For intraclass correlation we also used R Studio with package rptR and its function 'rpt()' (Stoffel et al., 2017), which estimates the repeatability of the two AUC measures by bootstrapping. We report the median bootstrapped distribution of the correlation between the two AUC measures per participant and their 95% confidence intervals.

## 3. Results

### 3.1 Sample Characteristics

Sociodemographic and participants' consumption data are presented in Table 1. We report age, sex, ethnic group, marital status, educational level, country of origin, monthly income, self-monitoring, intelligence, type of alcohol consumption and number of other consumption substances. Based on the AUDIT cut-off score, the majority of participants were classified as binge drinkers (M = 10.88; SD = 4.81). Categorical variables are expressed as percentages and divided according to the assigned group.

**Table 1:** Sociodemographic and Consumption Characteristics of the Participants

	Future-oriented Group (%)	Control Group (%)
Age		
18 - 25	90.7	95.3
26 - 31	9.3	4.7
Sex		
Female	48.8	62.8
Male	51.2	37.2
Marital Status		
Single	97.7	100
Other	2.3	0
Educational Level		
University	100	100
Country Origin		
Mexico	100	100
Monthly Familiar Income		
<$5,200 mxn	11.6	11.6
$5,200 y $10,400 mxn	11.6	18.6
$10,401 y $15,600 mxn	37.2	25.6
$15,601 y $20,800 mxn	18.6	11.6
>$20,801 mxn	21.0	32.6
Self-monitoring		
Low	18.6	30.2
Medium	65.1	46.5
High	16.3	23.3
Intelligence		
Below Average	0	2.3
Average	27.9	37.2
Above Average	60.5	55.8
Well Above Average	11.6	4.7
Type of Alcohol Consumption		
Abuse	69.8	74.4
Low-Risk	30.2	25.6
Number of other consumption substances		
Substances		
	44.2	51.2
0	46.5	37.2
1 Tobacco	85	50
1 Cannabis	15	44
1 Other	0	6
	9.3	11.6
2 Tobacco and Cannabis	75	60
2 Other	25	40

*Note. n = 86 (43 participants in each group). The category “Other” includes substances such as sedatives and hallucinogens, which were the most frequently reported after tobacco and cannabis.*

### Delay Discounting and Future Thinking Priming Effect

The 81% of the delay discounting functions were systematic and the remaining 19% nonsystematic. Of the nonsystematic functions, 27.27% met Criterion 1, 69.7% Criterion 2, and 3.03% both. Most of nonsystematic data appeared in the post-test measurement (64%) and in the control group (61%).

**Figure 1:**
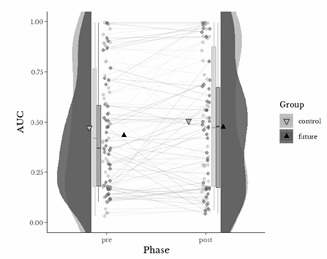
AUCs Pre- and Post FTP of all Participants

*Note. The dots on the graph represent the pre- and post-test AUC values for each participant (n = 86). Each pair of dots is connected by a line, with the line color corresponding to the participant's group (control or future). The box plots show the interquartile range (Q1-Q3), with a line inside each box representing the median; outliers are shown as individual points. The violin plots illustrate the overall distribution of the data, with pre-test AUCs on the left and post-test AUCs on the right. The black triangles mark the pre- and post-test AUC means for the future-oriented group, and the gray inverted triangles mark the corresponding means for the control group*

Figure 1 shows AUC pre and post FTP of all participants along with the distribution, mean and median of AUC of both groups in both phases. The dark gray color represents the dataset for the future-oriented group, while the light gray color represents the dataset for the control group. The X axis represents the phases associated with the two discounting measurements, while the Y axis displays the corresponding AUC values. There was no significant reduction in participants' delay discounting after FTP administration. Our main model showed a negative and statistically non-significant group fixed effect (3 = -0.04, 95% C.I. -0.17 to 0.09, *p* = 0.62), indicating no significant difference between mean AUC of future-oriented and control group. This model also showed non-significant fixed effects of phase (3 = 0.03, 95 % C.I. -0.03 to 0.09, *p* = 0.44), nor interaction (3 = 0.009, 95 % C.I. -0.07 to 0.09, *p* = 0.87), which means that there were no substantial general differences between the pre and post-test measures of discounting in both groups.

**Figure 2:**
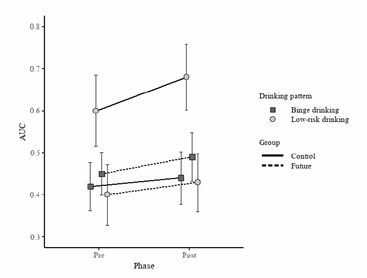
Mean of AUC of Future Oriented and Control Group Based on Type of Alcohol Consumption

Figure 2 shows the mean of AUC of both groups in both phases based on the type of alcohol consumption. Black squares represent the AUC means (pre and post) for the participants classified as binge drinkers, while the gray circles represent the AUC means (pre and post) for the participants classified as low-risk drinkers. The participants in the control group are represented with a solid line, and those in the future-oriented group with a dashed line. The X axis illustrates the two discounting measures, while the Y axis indicates AUC values. Likewise, we did not find a significant effect of FTP in relation to type of alcohol consumption. The model incorporating alcohol as a factor indicated no statistically significant interactions between phase and type of consumption (3 = 8.1e- 4, 95% C.I. -0.01 to 0.01, *p* = 0.90); group and type of consumption (3 = 0.01, 95% C.I. -0.01 to 0.03, *p* = 0.39), nor between phase, group and type of consumption (3 = 4e-3, 95% C.I. -0.02 to 0.01, *p* = 0.64). In other words, FTP was not associated with lower discount rates in either participants with al cohol abuse or those with low-risk drinking.

### 3.3 Intraclass Correlation of Delay

Finally, we obtained a high intraclass correlation repeatability of both discounting measures. The median bootstrapped distribution between the pre and post AUC mean of each participant was 0.80 with 95% C.I. [0.72 to 0.86] differing significantly zero. These results indicate that the delay discounting measures of participants remained notably stable throughout the two-week period of their participation (for further illustration of each delay discounting measure, see Figure 1).

## 4. Discussion

The aim of this study was to evaluate the effects of the Future Thinking Priming (FTP) on individuals engaging in binge and low-risk drinking. Results suggest that FTP failed to reduce delay discounting, regardless of participants' type of alcohol consumption. This result differs from previous studies where FTP significantly reduced discounting (Sheffer et al., 2016; Shevorykin et al., 2019, 2021). On the other hand, we found a strong and significant correlation between pre and post-test measures of delay discounting for all participants.

The correlation observed in delay discounting measures aligns with previous research emphasizing the stability and reliability of these measures. For instance, a recent me- ta-analysis by Gelino et al. (2024) reported an average test-retest reliability coefficient of r = 0.67, 95% C.I. [0.618 to 0.716]. Despite differences in statistical methods, both our study and this meta-analysis exhibit comparable confidence intervals and high consistency across delay discounting measures, in line with earlier findings (e.g., Black & Rosen., 2011; Kirby, 2009). Our findings robustly support previous reports that discounting measures remain stable under controlled conditions (Landes et al., 2012). Furthermore, they may support the conceptualization of delay discounting as a trait-like variable (e.g., Odum et al., 2020; Rung et al., 2019; Cassidy et al., 2020) and con tribute to broader discussions on the extent to which discounting is modifiable and how such modifications influence individual's decision-making beyond laboratory settings (Athamneh et al., 2021).

Regarding the nonsignificant FTP effect, our study's smaller sample size (15-19 times smaller than Sheffer et al., 2016; Shevorykin et al., 2019) limits our ability to precisely estimate the effect size and detect small effects. While our study was adequately powered for the expected effect size, the confidence intervals around our FTP effect estimate are wider than those from larger studies. This reduced precision makes it difficult to distinguish between a true null effect and a small effect that our sample size was insuffcient to detect reliably.

In larger samples the effect of FTP appears to be considerably small. For example, while Shevorykin et al. (2019) reports statistically significant differences in discounting be- tween groups, the differences were only at the threshold of statistical significance (*p* = 0.05), even with 1532 participants, and the effect size was also small. Our power analysis may have assumed an effect size larger than what actually exists for this phenomenon. Such small effects, although significant, may lack practical relevance in reducing substance use. If the goal is for the intervention to yield practical effects, it would be more effective to focus on strategies that enhance its effect size, rather than simply increasing the sample size. Given these considerations and our statistical power analysis, the sample size may not be the main explanation for the absence of the FTP effect; rather, the issue may be that the typical effect size for this phenomenon is smaller than assumed.

Another possible explanation may be the Spanish translation of future-oriented words. A few participants did not associate the word “restraint” (i.e., contención in Spanish) with self-control or the future. For example, one participant applied the term within the context of a soccer game: *"in soccer games, my position is of restraint"*, meaning that their usual position in soccer is a defensive midfielder. Although we tried to minimize differences in word meanings by translating them with the help of Spanish speakers who are native English speakers, it is possible that the simple translation was insufficient to trigger a greater preference for future rewards. Additionally, it must be considered that priming effects vary based on individual's experiences with the stimuli to which they are exposed (Cesario, 2014; Ramscar, 2016).

We observed a wide range of experiences associated with the future-oriented words. Importantly, some of the participants as- sociate these words with a negative affect (e.g., *"I get frustrated thinking about the future"*; *"I'm afraid of the future"*) or negative experiences (e.g., *"I used to have good willpower until I realized I was holding back from living my life"*; *"saving time has been counterproductive in my life"*); others see them as obstacles (e.g., *"I realized that dis cipline and self-control were obstacles that prevented me from having satisfying experiences"*; *"saving is an obstacle for me"*); while others associate them with more positive affects (e.g., *"I like planning things so that they turn out as I expect"*; *"I like doing my activities with discipline"*). It is possible that the positive or negative affects attributed to the words in the sentences interfered with some of the participants' response to the FTP, especially considering that negative affects are associated with greater delay discounting and positive affects with reduced discounting (Malesza, 2021; Rung & Madden, 2018; Scholten et al., 2019). Future studies should explore with appropriate statistical power whether the positive or negative affects of the words in the sentences moderate participants' response to the FTP.

It should also be considered that a priming effect is unlikely to trigger behaviors outside of an individual's repertoire (Weingarten et al., 2016). In the self-referential sentences, many participants did not attribute themselves behaviors related to self-control, discipline, or future-oriented actions (e.g., *"I don't consider myself a very disciplined person"*; *"I'm not much of a future thinker"*). Other participants mentioned that achieving such behaviors was difficult (e.g., *"I struggle to have discipline"*; *"I find it hard to be patient when offered something long-term"*) and some emphasized this difficulty in relation to alcohol consumption (e.g., *"I lack self-control when drinking; when I'm sad, I can't restraint the urge to drink"*). These self-referential sentences express the participants' capacity in performing self-controlled or future-oriented behaviors, which may be related to their self-efficacy.

Self-efficacy refers to individuals' belie- fs of their capacity to execute behaviors needed for a specific performance or the control of situations (López-Torrecillas et al., 2002). Individuals with substance misuse exhibit low levels of self-efficacy, and evidence suggest that the lower the self-efficacy, the greater the likelihood of maintaining substance use and experiencing relapses (Athamneh et al., 2019). Moreover, self-efficacy is related to delay discounting levels in people with substance abuse (Athamneh et al., 2019; Sheffer et al., 2012) and can influence motivation levels as well as the achievement of specific goals associated to behavioral changes (López-Torrecillas et al., 2002). Therefore, it is possible that the sentences reflect the participants' self-efficacy regarding the execution of future-oriented or self-controlled behaviors. We recommend that future studies on FTP include self-efficacy as a moderator of its effect, especially in populations dealing with substance abuse.

One last explanation for the failure to obtain a significant effect of FTP is the general limitations related to priming effects, specifically their low replicability (Cesario, 2014), and the evidence of their marginal effect on behavior (Weingarten et al., 2016). In this context, the probability of a priming effect easily changing the pattern of intertempo ral choices is low (Lippert & Suurmets, 2021; Rung & Madden, 2018) which could potentially explain the modest effect of FTP in studies with larger samples. However, it has been argued that the modest priming effects are due to unidentified moderating variables (Cesario, 2014; Sherman & Rivers, 2021) and at present, the moderators of the FTP are unknown, which highlights the need to identify them. We believe that the content of self-referential sentences may provide relevant information about these potential moderators. Furthermore, we highlight the importance of future studies considering these limitations of priming effects in their evaluations and potential applications of the FTP.

This study has some limitations that should be acknowledged. First, a minority of participants (12%) reported medication use, this variable, and the type of substance used -which were not controlled-may impact the performance on the DD tasks (e.g., Sarmiento et al., 2023; Amlung et al., 2017). However, implementing such controls could have limited the statistical power of our analyses. Second, the classification of participants' drinking patterns relied on the AUDIT cut-off score (> 8), which may have resulted in a narrow margin between low- risk and binge drinkers, potentially reduced the sensitivity of group comparisons. Future studies could consider adopting higher cut-off scores in the AUDIT to increase this sensitivity. Lastly, contex tual factors such as the predominant type of alcoholic beverage consumed and the unequal distribution of men and women across groups may have introduced additional variability into the results.

## 5. Conclusión

This study did not find evidence that FTP reduces delay discounting among individuals with binge or low-risk drinking. The non-significant effect of the intervention may be explained by several factors, including its small effect size reported in previous studies and the broader limitations of priming effects. Importantly, the self-referential sentences may reveal potential moderators of FTP, such as self-efficacy and affective valence, which should be addressed in future research, particularly in populations with substance use problems. Finally, exploring strategies to enhance the effect size of FTP may help clarify its role in reducing delay discounting and, ultimately, its practical relevance for interventions targeting substance use.

## Appendix A. General Instructions of De- lay Discounting Task

“Thank you for participating in this experiment. Your participation is very important. On the following screens, you will be presented with a series of questions and asked to answer as you would in real life. Imagine the following:

If you had the opportunity to win only one of two amounts of money and had to choose between a *large amount after a certain time* or a *small amount immediately*, what would you prefer? Would you prefer to wait for the large amount, or would you prefer to receive less but immediately? Next, diverse situations like this will appear. Each situation will be different as the amount and the time will change, *so NO situation is related to another*. There are no right or wrong answers. Your only job is to answer *as if each question was a real and unique situation*. Each question is independent of the previous and following ones.

ANY QUESTIONS? When you are ready to begin, press continue.”

## Appendix B. General Instructions of FTP

General Instructions

“Please read aloud each of the words that will be presented to you next. Make sure to copy each one onto a sheet of paper before proceeding. In the following steps, you will need to use each of these words, and you will not see the list again.”

Sentences Instructions

"In the next section, you will be asked to write a total of 10 different sentences describing yourself using the words from the list you just read and copied. Each sentence must include at least one of the words on the list. Make sure that in each sentence you use only pronouns that describe you, such as I, me, my, mine, ... etc. Example, if one of the words is "worried", the sentence can be "I am worried..." or if the word is "fun" the sentence can be "I am fun...". If you write, for example "my friends are fun" even if you use the pro- noun my, the sentence describes your friends and not yourself, and it is imperative that the sentences refer only to you. You will be presented with a separate box to write each sentence. Once you have finished writing the first sentence you will click on the next button to write the second sentence and so on until you have completed all 10 sentences. When you have completed these 10 sentences, you will click the submit button."

Paragraph Instructions

"You will now write a short paragraph des- cribing yourself using the same 10 words that were presented to you earlier. Again, these words will have to describe you and no one else. Please make sure that each of the 10 words on the list are included within your paragraph. The sentences you include here cannot repeat or be the same as the ones you wrote in the previous exercise. Your paragra- ph cannot be longer than 250 words."
